# Safety and Efficacy of Intracameral Methotrexate and Targeted Radiotherapy for Subconjunctival Ocular Granulocytic Sarcoma: A Case Report

**DOI:** 10.7759/cureus.81351

**Published:** 2025-03-28

**Authors:** Arkadiusz Chmielewski, Anna Machalinska, Slawomir Milczarek, Boguslaw Machalinski

**Affiliations:** 1 Ophthalmology, Pomeranian Medical University, Szczecin, POL; 2 Hematology and Transplantology, Pomeranian Medical University, Szczecin, POL

**Keywords:** acute myeloid leukemia, confocal microscopy, corneal endothelium, intracameral methotrexate, ocular granulocytic sarcoma, radiotherapy

## Abstract

A 28-year-old male patient with a history of acute myeloid leukemia, who was in complete remission for 18 months after allogeneic peripheral blood stem cell transplantation (PBSCT), developed a salmon-pink nodular lesion of the upper bulbar conjunctiva with conjunctival vascular engorgement of the right eye, accompanied by severe anterior uveitis and hypopyon. Ocular granulocytic sarcoma (OGS) was diagnosed based on immunohistochemistry analysis of the tissue sample and flow cytometry analysis of the aqueous humour, representing a local recurrence of acute myeloid leukemia. Systemic and central nervous system infiltration was excluded. Targeted radiotherapy of the right eye (24 Gy/12 cycles) and a series of 12 intracameral injections of methotrexate (400 μg/0.1 ml) were implemented with no impact on the endothelial cell count or corneal morphology. Complete macroscopic and cytometric remission of the lesion was achieved without leaving any permanent visual defects.

## Introduction

The case of ocular granulocytic sarcoma presented here is one of the few that has been described in the literature. The subconjunctival localization of the lesion makes it even more unique. Among the reported cases, the disease has occurred at different stages of acute myeloid leukemia (AML). In some patients, it preceded the systemic manifestation of AML by years; in others, it coexisted with it or developed years after AML systemic remission [1]. Myeloid sarcoma (MS) was first described in 1811 and was called “chloroma” because of the green colour of the lesion, which is caused by the myeloperoxidase content of the cells [2]. In 1966, owing to the great variability in the macroscopic appearance of the lesion (as not every lesion is green), it was renamed granulocytic sarcoma (GS) [3].

## Case presentation

**Figure 1:**
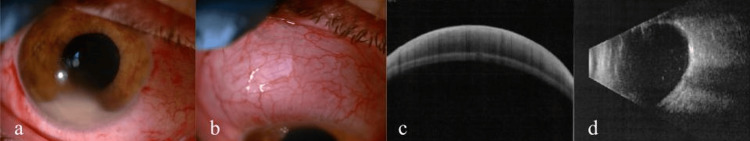
Initial state on admission to the hospital: exudate in the anterior chamber (a) and subconjunctival lesion in a slit lamp (b), SS-OCT scan through the subconjunctival lesion (c), B-scan ultrasonography (d) SS-OCT - swept source optical coherence tomography

A 28-year-old patient with anterior uveitis of the right eye was referred to our clinic. The patient had undergone rescue allogeneic peripheral blood stem cell transplantation (PBSCT) after total body irradiation-based conditioning for FLT3-ITD acute myeloid leukemia 18 months earlier. The procedure resulted in complete morphological remission with negative measurable disease. On admission to our hospital, the patient reported significant deterioration of vision and pain in the right eye. Best-corrected visual acuity was 0.2, without improvement with ocular correction. Increased intraocular pressure (33 mmHg, measured with Goldmann applanation tonometry), keratic precipitates, and white infiltrate in the anterior chamber of the right eye were found (Fig. 1a). In addition, a ciliary injection, corneal edema, and a hard, immovable, and nontender salmon-pink lesion localized in the subconjunctival tissue were present (Fig. 1b, 1c). Ophthalmoscopy was difficult to perform because of the reduced transparency of the media. There was no inflammatory exudate in the vitreous chamber on B-scan ultrasonography (Fig. 1d). During hospitalization, treatment with topical moxifloxacin (0.5%), dexamethasone (0.1%), timolol (0.5%), brimonidine (0.2%), oral acetazolamide (1000 mg/d), and subconjunctival injections of biodacin with dexamethasone were implemented. A sample of aqueous humour from the anterior chamber for flow cytometry and bacterial culture was taken. A biopsy of the subconjunctival lesion for histopathological examination was performed. After partial improvement of the patient's eye condition, he was discharged and redirected for further outpatient care.

During follow-up visits at the outpatient clinic over the next month, gradual deterioration of the patient's eye condition was observed. The pain in the right eye intensified, and the amount of white exudate in the anterior chamber increased. The subconjunctival lesion had grown, and the intraocular pressure values remained high (32-37 mmHg) despite the use of topical and oral antiglaucoma drugs. The bacterial culture and smear of the aqueous humour were negative. Topical steroids were continued, and oral steroid therapy with methylprednisolone (48 mg/d) was implemented. Worsening corneal edema and a reduced best-corrected visual acuity up to 0.5/50 were observed.

Histopathological examination with immunohistochemical staining of the sample of the subconjunctival lesion revealed the presence of mononuclear cells in the connective tissue, indicating the expression of MPO+, CD33+, CD15+, and CD34+, which is consistent with blasts of myeloid origin. Cells with HLA-DR+, CD33+, and CD13+ expression in aqueous humour were identified. Local recurrence of acute myeloid leukemia in the form of ocular granulocytic sarcoma (OGS) with subconjunctival localization was diagnosed. The patient was referred to the hemato-oncology department for further systemic evaluation. Based on MRI of the brain, lumbar puncture, and bone marrow biopsy, systemic recurrence of AML was ruled out.

In collaboration with the hemato-oncology department, a treatment plan for the patient was established. Targeted radiotherapy of the subconjunctival lesion with a total dose of 24 Gy in 12 cycles followed by 12 intracameral injections of methotrexate (400 μg/0.1 ml) was planned. Injections were projected on the following schedule: one injection per week for the first month, followed by one injection every 2 weeks for the second month, and successively one injection per month for 6 months, for a total of 12 injections.

**Figure 2:**
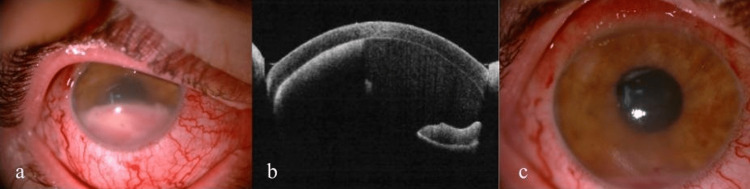
Condition of the eye after two initial irradiations: slit lamp examination showing the increased amount of exudate in the anterior chamber (a), AS-OCT of the anterior chamber (b), slit lamp image showing improved condition of the right eye after anterior chamber irrigation (c). AS-OCT - anterior segment optical coherence tomography

During radiotherapy, the patient visited the outpatient clinic for regular ophthalmological check-ups. Topical and oral steroids and antiglaucoma drugs were maintained. Dexamethasone 3 mg/d, instead of methylprednisolone 48 mg/d, was used. After the first two irradiations, the condition of the eye deteriorated even further. The amount of exudate in the anterior chamber increased significantly. The intraocular pressure rose up to 46 mmHg, corneal edema worsened, central corneal thickness (CCT) increased up to 675 μm, the pain intensified, and visual acuity deteriorated to light perception (Fig. 2a, 2b). Eyelid edema and redness were observed. A decision was made to irrigate the accumulated exudate from the anterior chamber of the right eye. After irrigation, the condition of the eye improved significantly (Fig. 2c). The pain, intraocular pressure, and corneal edema decreased. Oral acetazolamide (1000 mg/d) was discontinued, and oral dexamethasone (3 mg/d) in conjunction with topical steroids and antiglaucoma drugs was maintained.

**Figure 3:**
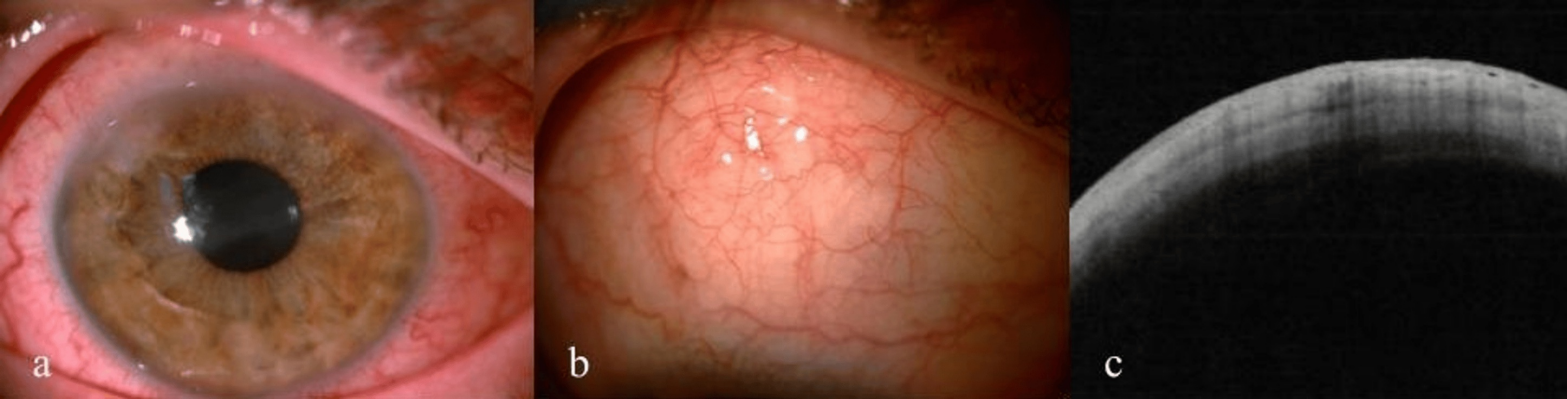
Condition of the eye after completing radiotherapy: slit lamp examination of the anterior chamber (a) and subconjunctival lesion (b), SS-OCT scan through the lesion (c). SS-OCT - swept source optical coherence tomography

After completing targeted radiotherapy (24 Gy/12 cycles), macroscopic remission of the sarcoma and no recurrence of the exudate in the anterior chamber were observed (Fig. 3). However, the condition of the eye adnexa and its surface deteriorated. Eyelid edema, periocular erythema, loss of eyebrow hair, and loss of eyelashes appeared. The conjunctiva was severely injected, with initial scarring. Mucous filaments on the cornea and corneal punctate epitheliopathy were present. A diagnosis of severe keratoconjunctivitis sicca was made. Intense therapy with artificial tears with trehalose and sodium hyaluronate and warm compresses was implemented. Topical steroids and antiglaucoma drugs were maintained.

**Figure 4:**
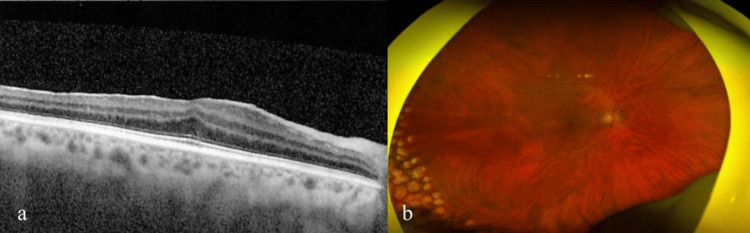
Retinal state after radiotherapy and 12 intracameral injections of methotrexate: OCT scan showing the epiretinal membrane (a) and atrophic lesions on the far temporal periphery (b). OCT - optical coherence tomography

**Figure 5:**
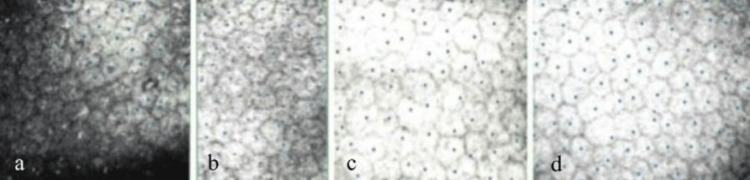
Confocal microscopy images of the corneal endothelium after 2 (a), 4 (b), 8 (c) and 12 (d) intracameral methotrexate injections.

Next, a series of 12 intracameral injections of methotrexate (400 μg/0.1 ml) was implemented. The detailed morphology, number of endothelial cells, and the pachymetry map were verified using confocal microscopy and anterior segment optical coherence tomography before and at the time of each intracameral injection. Control flow cytometry analysis of the aqueous humour was performed approximately every two to three injections. Over the course of the treatment, successive improvements in the patient's eye condition were observed. Follow-up high-resolution swept-source optical coherence tomography (OCT) scans performed through the site of the lesion revealed no relapse of the sarcoma. Control flow cytometry analysis did not reveal the presence of leukemic cells in the aqueous humour. Corneal edema decreased with an average CCT of 511 μm. Eyelashes and eyebrow hair grew back, and the condition of the eye adnexa improved significantly. OCT scans through the macula and perimacular retina revealed the epiretinal membrane (stage 3 according to Govetto classification). The central retinal thickness (CRT) was 376 μm (Fig. 4a). Fundus examination revealed the presence of atrophic lesions of the retina on the far temporal periphery, which most likely represents a consequence of the applied radiotherapy (Fig. 4b). Evaluation of the endothelial cell status using confocal microscopy revealed no changes in their number or morphology after two, four, eight, and 12 intracameral injections of methotrexate (Fig. 5).

**Figure 6:**
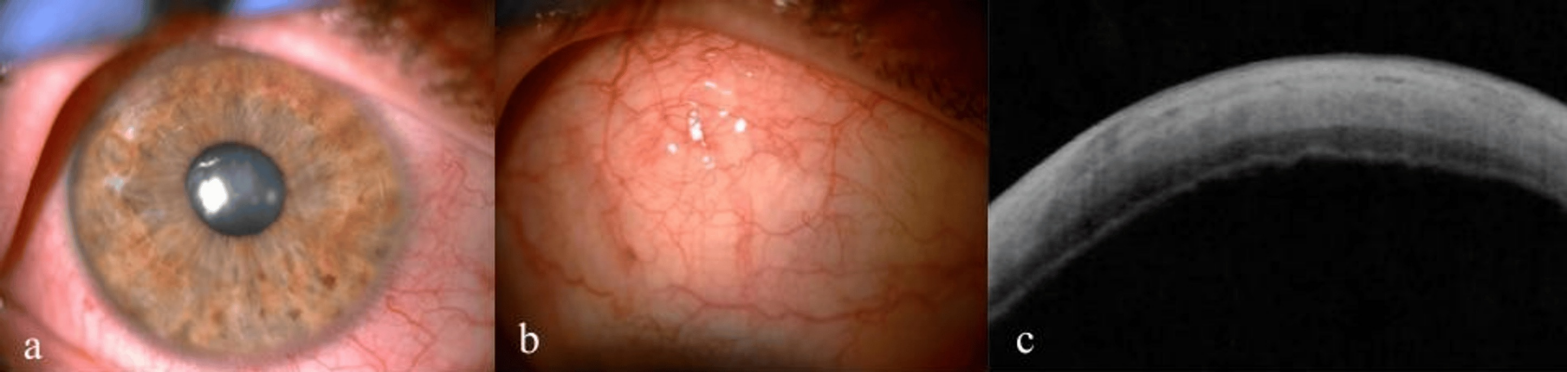
Final condition of the eye after radiotherapy and 12 intracameral methotrexate injections: slit lamp examination of the anterior chamber (a) and subconjunctival lesion (b), SS-OCT scan through the lesion (c). SS-OCT - swept source optical coherence tomography

At the end of the treatment (after 12 intracameral injections of methotrexate and 12 cycles of targeted radiotherapy), the average number of endothelial cells was 2967 cells/mm2, and the CCT was 531 μm. The patient’s best-corrected visual acuity was 0.8. The intraocular pressure normalized to 16 mmHg (Fig. 6). The patient remains under the care of the hemato-oncology department, where no systemic recurrence of AML has been reported to date.

## Discussion

Granulocytic sarcoma is a rare disease that occurs in 2.5-9.1% of AML patients. It is characterized by the presence of one or more extramedullary lesions in tissues, such as bones, subcutaneous tissue, orbits, lymph nodes, the digestive system, and the central nervous system (CNS) [4]. It is far more common in the pediatric population, where the skin and orbits are the most frequent locations. The most typical symptom is unilateral exophthalmos; other possible signs include ptosis, painful swelling of the lacrimal gland, a conjunctival mass, retinal hemorrhages, iris infiltration or choroidal lesions [5].

A few similar cases have been reported in the literature to date. For example, in a 65-year-old patient with isolated granulocytic sarcoma in the right eye conjunctiva diagnosed two years after remission of AML, only targeted electron beam radiotherapy was applied to the conjunctival lesion, resulting in permanent withdrawal of the tumor [6]. In a reported case of a 73-year-old patient suffering from myeloproliferative syndrome, bilateral ocular conjunctival granulocytic sarcoma was diagnosed. The patient underwent targeted radiotherapy of the conjunctival lesions with a total dose of 30 Gy along with systemic chemotherapy, resulting in complete resolution of the lesions [7]. In another case of a 50-year-old patient diagnosed with myeloid sarcoma in the left superior conjunctiva with systemic relapse of AML, reinduction chemotherapy with concomitant allogeneic peripheral blood stem cell transplantation and salvage radiotherapy (24 Gy/12 cycles) for the conjunctival lesion were implemented, resulting in total remission of the sarcoma [8]. Notably, the presented case is the only one documenting symptoms of anterior uveitis with pseudohypopyon in the course of granulocytic sarcoma, which has not been described in similar cases. According to the available data, hypopyon and severe intraocular inflammation have been reported in patients with extranodal natural killer/T-cell lymphoma [9] or chronic myeloid leukemia [10, 11].

We observed deterioration of the eye adnexa status and eye surface as a result of targeted radiotherapy. Indeed, keratoconjunctivitis sicca remains the most prominent side effect of radiotherapy. Symptoms and signs of dry eye disease with superficial punctate keratitis have been previously described in a similar case after radiotherapy with diffuse meibomian gland dropout [8].

We also reported the formation of an epiretinal membrane in the macular region. A similar effect has been documented in a child who underwent radiation therapy for craniopharyngioma, with epiretinal membrane formation as a consequence of exposure to low-dose radiation [12]. Epiretinal membranes have been previously observed in patients who underwent plaque radiotherapy [13] and stereotactic radiosurgery [14] for uveal melanoma.

Methotrexate in the form of intravitreal injections has already been used in ophthalmology. At a concentration of 400 μg/0.1 ml, it has been successfully used for the treatment of vitreoretinal lymphomas with good tolerance [15]. Methotrexate has been utilized as an adjunctive treatment for proliferative vitreoretinopathy in the course of retinal detachment because of its ability to inhibit cell proliferation and fibrosis and its anti-inflammatory properties [16].

There are only a few reports documenting the use of intracameral injections of methotrexate. At a dose of 400 μg/0.1 ml, it has been administered for epithelial ingrowth after Ahmed valve implantation, leading to permanent withdrawal of the ingrowing epithelium after 11 injections [17]. Accordingly, intracameral methotrexate has been used for treating leukemic glaucoma in the course of chronic myeloid leukemia, indicating that it might be helpful in eradicating blast cells from the aqueous humour and in controlling intraocular pressure [18].

Contrary to our data, the authors of the aforementioned studies did not monitor endothelial cell status or corneal parameters and therefore did not provide data regarding the safety profile of the applied therapy. To our knowledge, this is the first report of intracameral injections of methotrexate (400 μg/0.1 ml) in the case of OGS. The treatment regimen of 12 targeted irradiations at a total dose of 24 Gy with 12 intracameral injections of methotrexate (400 μg/0,1 ml) resulted in very good outcomes and has shown excellent safety profiles.

A detailed evaluation of the endothelium via confocal microscopy allowed us to perform a thorough analysis of the quantity and quality of endothelial cells, confirming that there was no impact of the applied therapy on cell morphology or number. This demonstrates the safety of intracameral injections of methotrexate at a concentration of 400 μg/0.1 ml. These valuable data can be used in similar cases and represent an interesting topic for further studies on a larger group of patients.

## Conclusions

Patients suffering from acute myeloid leukemia require special attention when alarming ocular symptoms appear. Granulocytic sarcoma represents a rare condition that, if untreated, can lead to loss of the eye and systemic spread of the disease. The diagnosis and treatment regimen used in this reported case was successful. Intracameral injections of methotrexate have shown a high safety profile with no impact on the endothelial cell count or corneal parameters.
